# Trans- and Multigenerational Effects of Isothiazolinone Biocide CMIT/MIT on Genotoxicity and Epigenotoxicity in *Daphnia magna*

**DOI:** 10.3390/toxics11040388

**Published:** 2023-04-20

**Authors:** Jiwan Kim, Jinhee Choi

**Affiliations:** School of Environmental Engineering, University of Seoul, 163 Seoulsiripdae-ro, Dongdaemun-gu, Seoul 02504, Republic of Korea; gimjiwang@gmail.com

**Keywords:** isothiazolinone, aquatic toxicity, transgenerational effect, multigenerational effect, genotoxicity, epigenotoxicity

## Abstract

The mixture of 5-chloro-2-methylisothiazol-3(2H)-one and 2-methylisothiazol-3(2H)-one, CMIT/MIT, is an isothiazolinone biocide that is consistently detected in aquatic environments because of its broad-spectrum usage in industrial fields. Despite concerns about ecotoxicological risks and possible multigenerational exposure, toxicological information on CMIT/MIT is very limited to human health and within-generational toxicity. Furthermore, epigenetic markers altered by chemical exposure can be transmitted over generations, but the role of these changes in phenotypic responses and toxicity with respect to trans- and multigenerational effects is poorly understood. In this study, the toxicity of CMIT/MIT on *Daphnia magna* was evaluated by measuring various endpoints (mortality, reproduction, body size, swimming behavior, and proteomic expression), and its trans- and multigenerational effects were investigated over four consecutive generations. The genotoxicity and epigenotoxicity of CMIT/MIT were examined using a comet assay and global DNA methylation measurements. The results show deleterious effects on various endpoints and differences in response patterns according to different exposure histories. Parental effects were transgenerational or recovered after exposure termination, while multigenerational exposure led to acclimatory/defensive responses. Changes in DNA damage were closely associated with altered reproduction in daphnids, but their possible relationship with global DNA methylation was not found. Overall, this study provides ecotoxicological information on CMIT/MIT relative to multifaceted endpoints and aids in understanding multigenerational phenomena under CMIT/MIT exposure. It also emphasizes the consideration of exposure duration and multigenerational observations in evaluating ecotoxicity and the risk management of isothiazolinone biocides.

## 1. Introduction

As biocides are extensively applied in various industrial fields, multiple biocides have been frequently detected in wastewater influent and effluent, sludge, surface water, and sediments [1,2]. Among the biocides, isothiazolinones are commonly used to control microbial growth and biofouling due to their effectiveness and fast-acting traits, and their use has increased over recent years [3,4]. A mixture of 5-chloro-2-methylisothiazol-3(2H)-one and 2-methylisothiazol-3(2H)-one in a 3:1 ratio (CMIT/MIT) is a powerful isothiazolinone frequently used in wastewater treatment, coatings, paints, and cosmetics as an active ingredient of the commercial biocide, Kathon [3,5,6,7]. CMIT/MIT is water soluble, and its high toxicity has been reported in freshwater and estuarine/marine organisms [8,9]. Furthermore, Paijens et al. reported that CMIT and MIT were included in prioritized biocides according to their use in urban areas, their emissions into runoff and receiving waters, and their hazards to aquatic organisms [10]. Although CMIT/MIT has high environmental risk potential for non-target species when released into aquatic systems, the available toxicity information is largely limited to human toxicity and in vitro studies.

Isothiazolinone biocides cause cellular growth inhibition and cell death resulting from the disruption of the central metabolic pathways of the cell by repressing several specific enzymes, the progressive loss of thiols from cysteine and glutathione (GSH), and the production of free radicals in the cell [4,5]. Indeed, the in vitro comet assay revealed a 7.5-fold higher olive tail moment than the control group in the lymphocytes of rats treated with CMIT [11]. In addition, CMIT/MIT caused the depletion of thiol, the consequential elevation in cytosolic Zn^2+^, and the generation of reactive oxygen species (ROS) in vascular smooth muscle cells [12]. These results suggest that CMIT and MIT might induce oxidative DNA damage, possibly linked to ROS. Some studies demonstrated that CMIT, MIT, and their mixture led to high levels of pro-apoptotic proteins and the release of pro-inflammatory cytokines via the regulation of the mitogen-activated protein kinase (MAPK) signaling pathway [4]. The MAPK pathway is involved in cell growth, endoplasmic reticulum (ER) stress, DNA damage, and inflammation. Accordingly, the possibility of genotoxicity and multigenerational effects of CMIT/MIT cannot be disregarded.

In the natural environment, organisms can be continuously exposed to various stressors, which may last for more than one generation, especially in short-lived invertebrates. In terms of environmental toxicology, multigenerational exposure, which reflects the exposure to pervasive pollutants in the environment, can cause cumulative damage, acclimation, or adaptation to particular chemicals over generations [13,14,15]. Although organisms are not directly exposed to environmental compounds, transgenerational toxicity can be induced by ancestral chemical exposure with the transmission of epigenetic hallmarks [16]. Despite the various exposure patterns that occur in the natural environment, traditional ecotoxicological assessments have rarely considered the long-term, multigenerational, and transgenerational effects of toxicants, such as endocrine-disrupting chemicals, on human and wildlife health [17,18].

The alteration of epigenetic markers has been suggested and used as a tool to identify chemical exposures [19]. However, the impact of environmental challenges on epigenetically mediated evolution and the transgenerational inheritance of phenotypes receives little attention compared with the epigenetic inheritance of diseases/pathologies [20]. A relatively large number of epigenetic studies have focused on mechanisms, diseases, and development within an organism’s life span (considered to be more representative of an intragenerational perspective) rather than on evolution and inheritance (considered to be more representative of a transgenerational perspective) [20].

*Daphnia* is an ecologically keystone species in aquatic ecosystems as a primary consumer and food source for planktivores and fish. The inherent phenotypic plasticity of daphnids enables them to overcome fast-changing environments, and such features can be helpful as a cornerstone for understanding the link between organisms and their environment [21] with regard to ecotoxicology. Under laboratory conditions, daphnids are maintained in their parthenogenetic state. During female parthenogenesis, recombination does not occur, and parthenogenetic offspring are genetically identical to their mother [22,23]. This reproductive trait makes *Daphnia* a model organism for studying epigenetic changes. Environmentally controlled multifarious polyphenism, phenotypic alterations, and sex determination were observed in genetically identical daphnids [23]. Furthermore, various environmental stressors alter global and gene-specific DNA methylation levels in daphnids, and these epigenetic alterations can support phenotypic responses to chemical exposure [23,24].

Considering the widespread use of CMIT/MIT and its continuous detection in surface waters [10], it is necessary to evaluate the impact of CMIT/MIT on aquatic ecosystems using keystone species over multiple generations. Herein, we investigated the toxicity of CMIT/MIT to *Daphnia magna* under two exposure scenarios (i.e., only parental exposure and recovery (PE) vs. exposure of four consecutive generations; multigenerational exposure (ME)) by measuring phenotypic biomarkers, DNA damage, and global DNA methylation over four consecutive generations.

## 2. Materials and Methods

### 2.1. Daphnid Cultures

The model organism *Daphnia magna* was originally provided by Korea Institute of Toxicology (Daejeon, Republic of Korea). Daphnids reared in our laboratory were used for the experiments. Sixty animals per liter were individually placed in glass beakers containing fully aerated M4 medium. Daphnids were fed the green algae *Chlorella* sp. at concentrations of 4 × 10^5^ cells/mL∙d and maintained at 20 ± 1 °C under a 16 h light–8 h darkness cycle photoperiod regime. The medium was replaced three times per week.

### 2.2. Exposure to Chemicals and Exposure Scenarios

CMIT/MIT was purchased from Sigma-Aldrich (PHR 1597-1ML, Merck/Millipore Sigma, St. Louis, MO, USA). The concentrations of CMIT and MIT in stock solutions were measured using high-performance liquid chromatography with diode array detection (HPLC-DAD) under specific conditions (Table S1). It was confirmed that the concentrations of CMIT and MIT were present in a ratio of 3:1 in standard solution and stock solutions (the stock solutions were prepared by diluting the standard solution, which had a concentration of 14,000 mg/L, with distilled water to obtain concentrations of 1.4 and 0.14 mg/L). The exposure concentrations in the test media were maintained for seven days at 20 °C after spiking (Table 1). Stock solutions were diluted with M4 medium and used as working solutions. We first examined the general ecotoxicity of CMIT/MIT using phenotypic biomarkers (mortality, reproduction, body size, morphology, and swimming behavior) and proteomic analysis on *Daphnia magna* within a single generation. To determine the appropriate concentration of CMIT/MT for multigenerational studies, we tested the mortality and reproduction assay and estimated the 20% effective concentration (EC_20_) to be used.

**Figure 1:**
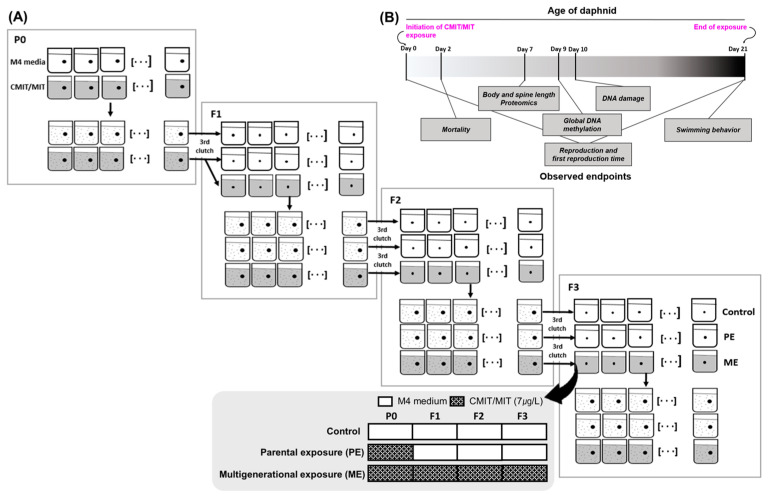
Experimental workflow for multigenerational studies: (**A**) Illustration of three different exposure designs (control, parental exposure, and multigenerational exposure) across four generations (P0: parental generation; F1: first filial generation; F2: second filial generation; F3: third filial generation). The third clutch of female daphnids was used for the next generation. (**B**) Detailed experimental schedules for the assessment of various endpoints within a single generation. Exposure was initiated with neonates on day 0 and ended on day 21.

The effects of chronic exposure to EC_20_ on reproduction and body size were then compared over four generations between the PE and ME scenarios. Finally, we investigated the multigenerational and transgenerational effects on DNA damage and global DNA methylation to identify whether genotoxicity and/or epigenotoxicity contributed to the modified phenotypes. Using the estimated chronic EC_20_ (7 µg/L) (Table S3), the toxicity of CMIT/MIT was examined across generations under three different exposure scenarios (Figure 1A). Neonates (<24 h) were exposed to M4 and CMIT/MIT solutions and then allowed to grow to 21-day-old adults for each generation. As described in the experimental workflow, all endpoints were assessed according to the scheduled dates (Figure 1B), and the third clutch of female daphnids in each generation was transferred into a clean medium and CMIT/MIT solution to maintain the next generation. Hereafter, the scenario in which the organisms were exposed only in the first generation is referred to as parental exposure (PE), whereas the scenario in which the organisms were continuously exposed for four generations is referred to as multigenerational exposure (ME).

### 2.3. Mortality and Reproduction Assay

Prior to the acute toxicity test, several range-finding tests were conducted to determine the concentration range that caused mortality from 0 to 100%. The acute toxicity test was then performed according to OECD Test Guideline 202 [25] at the following concentrations: 0, 20, 40, 80, 160, and 320 µg/L. After 48 h of exposure to CMIT/MIT, dead and immobilized neonates were counted (20 animals per treatment; 5 animals per beaker; n = 4). The concentration of 80 µg/L, which showed an impact on mortality in the acute toxicity test, was selected as the highest concentration for the chronic toxicity test.

For the chronic toxicity evaluation, a reproduction assay was performed for 21 days by observing the number of neonates in each clutch and the time to reproduction (age at each reproduction) according to OECD Test Guideline 211 [26]. During the 21 d reproduction test, the daphnids were exposed in a glass beaker containing 100 mL of M4 medium or CMIT/MIT solution (n = 10 for each treatment: 0, 5, 10, 20, 40, and 80 µg/L). Daphnids were transferred to a new M4 medium or chemical solution three times a week.

Lethal concentrations (LCs) and effective concentrations (ECs) were estimated based on the number of dead neonates and the total number of neonates produced by an adult, respectively. Finally, the estimated EC_20_, 7 µg/L, was determined to be the appropriate concentration for behavior assays and multigenerational studies. EC_20_ is estimated to be an environmentally relevant concentration that minimizes adverse effects on a population and is also used as a common toxicity reference value in ecotoxicological studies.

### 2.4. Behavior Assay

Behavior recording, computational movement tracking, and parameter calculations were adopted from Lee et al. [27] with some modifications. Swimming behavior was assessed in 21-day-old daphnids after exposure to 7 µg/L CMIT/MIT as another chronic toxicity endpoint (Figure S1). The technical details of the components (e.g., transparent box and video recorder), image processing procedures using Virtual Dub software (http://www.virtualdub.org/ (accessed on 7 June 2022)), and image analysis using ImageJ software (National Institute of Health, Maryland, USA) and MATLAB software (MathWorks, R2017b, Natick, USA) are shown in the supporting information (Figure S1). Before recording for five minutes, all organisms were allowed to acclimate to the medium (M4 or CMIT/MIT) for ten minutes. The behavior of daphnids (n = nine or ten) was analyzed based on movement parameters, including path length (total x, y pixel distance), speed (mm/s), locomotory rate (mm/s), stop number (n), and turning rate (rad/s), using ImageJ and MATLAB.

### 2.5. Morphology and Body Size

A single organism from each test beaker (n = 10) on days 0 and 7 was placed on a glass slide. After removing the moisture around the daphnids, their morphology, body length, and spine length were analyzed under a microscope (MZ6; Leica Microsystems, Wetzlar, Germany). The daphnid was measured from the top of the head to the starting point of the tail spine for body length and from the starting point to the base of the tail spine for spine length using the LAS. V4 program.

### 2.6. Quantitative Mass Spectrometry (MS)-Based Proteomic and Bioinformatic Analyses

Proteomic analyses were conducted on live 7-day-old P0 adults from both culture conditions, that is, M4 and EC_20_ estimates of CMIT/MIT (7 µg/L) (Figure 1). We selected and pooled daphnids that did not harbor any eggs or neonates in their backs to avoid the effect of egg and neonate formation on protein expression (ten daphnids per tube; n = 3). Protein extraction and preparation, peptide labeling, mass spectrometry analysis, and the identification and quantification of proteins were performed as previously described [14]. The details of the method are provided in the supporting information. The bioinformatic analyses of protein–protein interactions and functional enrichments were performed in String 11.0 (https://string-db.org/ (accessed on 10 April 2023)) with selected proteins (the differentially regulated proteins between M4 and 21 d EC_20_ estimates of CMIT/MIT, >1.2-fold change).

### 2.7. Comet Assay

For the preparation of daphnids, 30 daphnids aged ten days were collected from the control and experimental tanks of each generation after exposure to 7 µg/L CMIT/MIT and pooled in a tube of 10 animals each (n = 3). An alkaline comet assay was performed using live organisms. Briefly, a slide precoated with normal agarose was spread with 100 µL of 1% low-melting-point agarose and allowed to solidify at 4 °C for five minutes. The cells were then lysed in a solution containing high salt and detergent (10 mM Tris, 100 mM EDTA, 2.5 NaCl, 10% DMSO, and 10% Triton X-100; pH 10), followed by incubation in unwinding/electrophoresis buffer (300 mM NaOH and 1 mM EDTA; pH > 13) at 4 °C for 20 min. For electrophoresis, an electric current of 300 mA (25 V) was applied for 30 min, and the slides were neutralized and dehydrated in 70% ethanol. The slides were then air-dried and stained with 50 µL of ethidium bromide (5 µg/mL). Approximately 30 cells per slide (three slides per treatment) were examined, and the olive tail moment was analyzed using a fluorescence microscope (Leica Microsystems, Germany) connected to the image analysis system Komet, version 5.5 (Kinetic Imaging Ltd., Nottingham, UK).

### 2.8. Global DNA Methylation Measurement

Thirty 9-day-old daphnids (ten animals per a tube; n = 3) for DNA methylation measurements were collected from the control and experimental tanks of each generation after exposure to 7 µg/L CMIT/MIT. Live animals in a tube were flash-frozen in liquid nitrogen and stored at −80 °C until DNA extraction. After homogenization, total DNA was extracted using a DNA extraction kit (NucleoSpin; Macherey-Nagel, Duren, Germany), and the concentration of the extracted DNA was qualified and quantified using a NanoDrop machine (NanoReady Touch; Life Real, Hangzhou, China). Global DNA methylation assays were then conducted using the MethylFlash global DNA methylation 5-mC ELISA Easy Kit (Epigentek, New York, NY, USA) according to the manufacturer’s instructions.

### 2.9. Statistical Analysis

Statistical analyses were performed using R version 4.0.4. If normality (assessed with the Shapiro–Wilk test) and equal variance (assessed with the Bartlett test) assumptions were met in the dataset, an independent t-test was performed. If normality and/or equal variance tests failed, we evaluated the statistical significance between the control and exposed groups using a nonparametric Wilcoxon rank-sum test. Significant differences among more than two groups were tested using the R software package nparcomp to perform nonparametric multiple comparisons. Dose-response curves and lethal/effective concentrations were estimated using a logistic model fitted with the least-squares optimization method using the “drc” package in R.

## 3. Results and Discussion

### 3.1. Environmental Concentrations of CMIT and MIT

The concentrations of CMIT/MIT detected in commercial products and aquatic environments were reviewed in the literature (Table S2). CMIT, MIT and CMIT/MIT mixture have been measured in various product samples, such as cosmetics, household products, and adhesive, suggesting the constant usage of CMIT and MIT until a recent date. The concentrations of CMIT and MIT in these products ranged from 3 to 35 µg/g and more than 10 to 60 µg/g, respectively. In addition, Alvarez-Rivera et al. [28] detected the CMIT/MIT mixture in cosmetic and household product samples at concentrations ranging from 0.095 to 67 µg/g.

Table S2 also shows concentrations in diverse aquatic environments of CMIT and MIT. The range of their environmental concentrations varies from ng/L up to µg/L levels according to media, weather, and sampling sites. However, Baranowska and Wojciechowska reported high levels of CMIT (5~11.57 µg/L) in river samples [29]. The concentration of MIT also could reach 0.35 µg/L in wastewater treatment plant effluent and 1.21 µg/L in untreated sewage according to some studies [30,31].

### 3.2. Acute and Chronic Toxicity of CMIT/MIT in Daphnia magna

**Figure 2:**
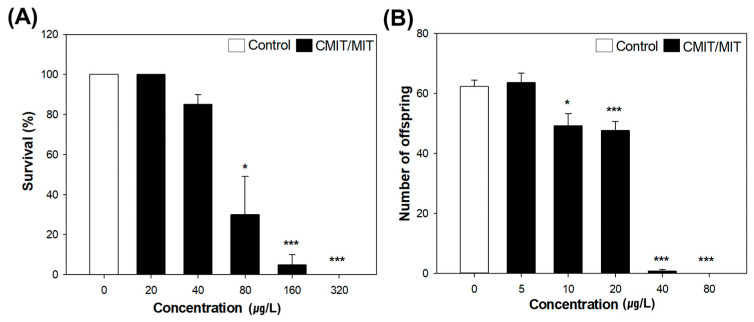
Effects of CMIT/MIT on mortality and reproductive capacity in *D. magna*: (**A**) Survival rate of daphnid neonates exposed for 48 h to 20, 40, 80, 160, and 320 µg/L CMIT/MIT. Data are presented as means ± SE (n = 4). (**B**) Total offspring numbers of adult daphnids exposed for 21 days to 5, 10, 20, 40, and 80 µg/L CMIT/MIT. Data are presented as means ± SE (n = 10). Asterisks indicate the significant differences between the exposure and control groups: * *p* < 0.05 and *** *p* < 0.001.

The mortality assay revealed that CMIT/MIT exposure decreases the daphnid survival rate in a concentration-dependent manner. Exposure to the concentrations above 80 µg/L significantly decreased the survival of daphnids. Among the test concentrations, no neonates survived at the highest concentration, that is, 320 µg/L (Figure 2A). Based on these results, the 48 h LC_50_ was estimated to be 63.20 µg/L (Table S3).

In addition, the results of the chronic test collectively suggest that CMIT/MIT possesses high reproductive toxicity to *D. magna*. The exposure to CMIT/MIT above 10 µg/L significantly reduced the total number of offspring for 21 days, but no significant difference was found in the first reproduction time (Figure 2B and Figure S2). The 21 d EC values were estimated and are presented in Table S3. The European Chemicals Agency (ECHA) previously reported the 21 d NOEC of CMIT/MIT (3.6 µg/L) for *D. magna*, which is comparable to 5 µg/L, the 21 d NOEC in this study [9]. However, the estimated 48 h LC_50_ in the present study was lower than that reported by the ECHA (100 µg/L).

**Figure 3:**
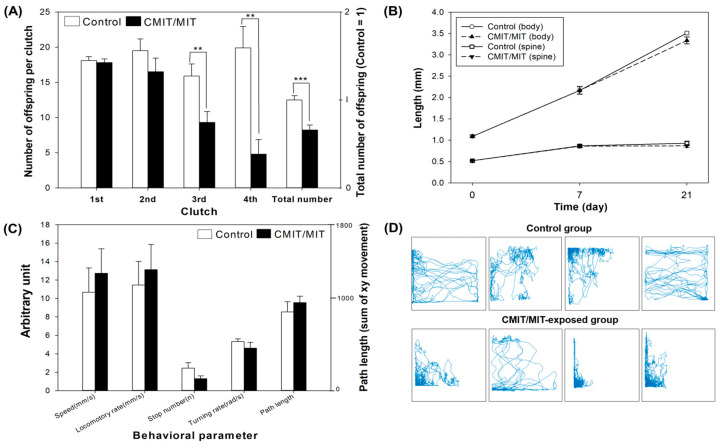
Reproductive capacity, growth, and swimming behavior of *D. magna* after exposure to EC20 CMIT/MIT (7 µg/L): (**A**) Number of offspring per clutch and total number of offspring produced by adult daphnids exposed to CMIT/MIT for 21 days. (**B**) Growth of daphnids was observed on days 0, 7, and 21 after exposure to CMIT/MIT. (**C**) The quantitative value of behavioral parameters (speed, locomotory rate, stop number, turning rate, and path length). (**D**) The representative pictures of two-dimensional pathway (four images were selected) in the control and exposure groups. Data are presented as means ± SE (n = 10), with the asterisks indicating significant differences between the exposure and control groups: ** *p* < 0.01 and *** *p* < 0.001.

Finally, we applied 7 µg/L CMIT/MIT mixture (CMIT: 5.25 µg/L; MIT: 1.75 µg/L; estimated according to Table 1), the concentration close to the chronic EC_20_, to all further experiments for the behavior assay and the multigenerational study. Aquatic organisms are plausibly exposed to this concentration, considering the detected concentrations in aquatic environments (Table S2). First, exposure to the chronic EC_20_ caused an adverse effect on the total reproduction (decreased by 34.2%), manifesting significant decreases in the third and fourth clutch sizes compared with the control group (Figure 3A), but no difference was observed in the first reproduction time (Figure S2). Second, the growth indicators were not affected by CMIT/MIT exposure (Figure 3B). The changes in spine length corresponded with the changes in body length. Third, all behavioral parameters did not show significant differences between the exposure and control groups (Figure 3C). However, we observed some changes in movement. The daphnids exposed to CMIT/MIT tended to stay at the edge of the test batch compared with the control group (Figure 3D).

### 3.3. Protein Expression Alteration in Response to CMIT/MIT Exposure

To gain an insight into the potential mechanism of the observed apical toxicity, a proteomic analysis was conducted on daphnids exposed to EC_20_ CMIT/MIT. From a total of 526 proteins, 132 differentially expressed proteins (DEPs; >1.2-fold change) in the P0 generation were identified in the CMIT/MIT-exposed groups with respect to the control groups. Among 132 DEPs, 69 proteins were upregulated, and 63 proteins were downregulated (Table S4). A total of 50 uncharacterized proteins were included in 132 DEPs.

**Figure 4:**
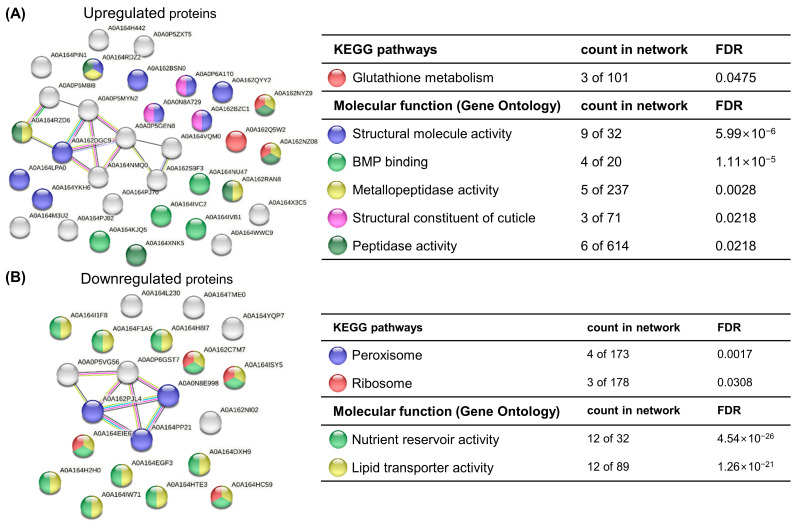
Protein–protein interaction networks and functional enrichments of differentially expressed proteins (DEPs) in *D. magna* after exposure to EC_20_ CMIT/MIT: KEGG pathways and GO molecular functions were enriched among (**A**) upregulated proteins and (**B**) downregulated proteins.

In the functional enrichment analysis, upregulated DEGs were enriched in KEGG pathways associated with glutathione metabolism and GO molecular terms associated with structural molecule activity, BMP binding, metallopeptidase activity, structural constituent of the cuticle, and peptidase activity (Figure 4A). Glutathione S-transferase (GST) delta protein (AOA162Q5W2), involved in glutathione metabolism, can be induced due to thiol depletion and ROS formation by CMIT/MIT [12]. GST is a biomarker, which indicates the exposure to xenobiotics and oxidative stress resulting in ROS production. Increased GST activity may also reflect GSH depletion during the detoxification processes due to the role of GST as a cofactor for glutathione peroxidase (GPx) and in catalyzing GSH conjugation to detoxify the chemicals [32].

The functional enrichment analysis of downregulated proteins showed peroxisome, ribosome, nutrient reservoir activity, and lipid transporter activity (Figure 4B). Peroxisomes are essential intracellular organelles that catalyze the decomposition of ROS to protect against oxidative stress and participate in various metabolic processes [33]. The downregulated proteins associated with peroxisomes can affect the decomposition of ROS produced by CMIT/MIT and cause reproductive toxicity. Furthermore, it is noteworthy that eight vitellogenin-related proteins are involved in lipid transporter activity (Figure 4B and Table S4). The downregulation of these proteins suggests a mechanism of reproductive impairment due to an effect on the reproductive system. Vitellogenin (VTG) is a major lipoprotein and a precursor of yolk protein vitellin (VTN), which vitally benefits the growth of embryos and reproduction by providing nourishment. Vitellogenin gene products in *D. magna* offer storage proteins that provide nutrients to the embryos during development, and the accumulation of vitellogenin in oocytes is one of the key events in the ovarian maturation process [34]. In addition, vitellogenin fused with superoxide dismutase (VTG1) is the most abundant polypeptide in the parthenogenetic eggs of *D. magna*, which might play a role in the immediate detoxification of superoxide resulting from vitellogenin metabolism or only work as a transporter of Cu in the domain [35]. Based on the decreased reproduction in the CMIT/MIT-treated daphnids, the proteomic analysis demonstrated the correlation between the downregulation of vitellogenin fused with superoxide dismutase proteins and reproductive failure. Therefore, we speculated that daphnids produced less eggs under CMIT/MIT exposure, since it takes longer to accumulate enough proteins to reach maturation.

### 3.4. Phenotypic Alterations under Parental and Multigenerational Exposure to CMIT/MIT: PE vs. ME

**Figure 5:**
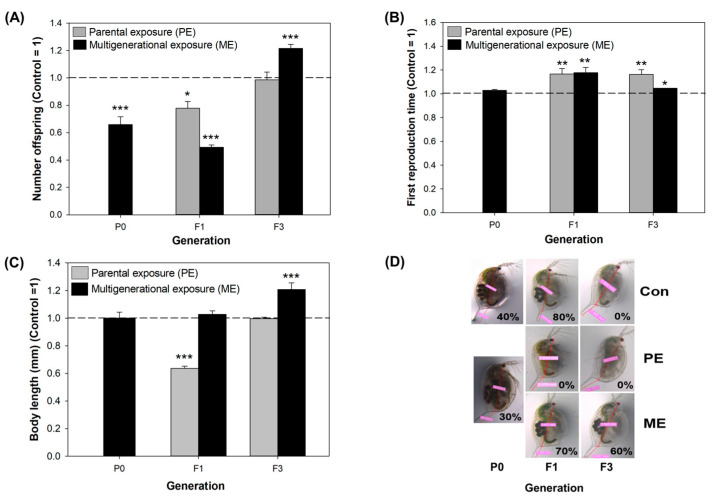
Phenotypic responses of daphnids to parental and multigenerational exposure to CMIT/MIT EC_20_. Data are normalized to the means of each control group and presented as normalized values ± SE (n = 10). Asterisks indicate significant differences between the exposure and control groups: * *p* < 0.05, ** *p* < 0.01, and *** *p* < 0.001. (**A**) Total number of neonates produced by adult daphnids. (**B**) First reproduction time of daphnids. (**C**) Body length of 7-day-old daphnid. (**D**) Representative images of the morphology and egg-holding rate (%) of daphnids under the two exposure scenarios. % indicates the percentage of organisms that had eggs or progenies in the brood chamber.

Rafoth et al. highlighted that CMIT and MIT in river water and tap water completely degraded within eight days and that the concentration decline at 4 °C significantly slowed [6]. Despite the short half-life, this duration is sufficient to affect the daphnid populations over multiple generations. Therefore, after confirming the significant adverse effects of CMIT/MIT on *D. magna*, we investigated the trans- and multigenerational effects of CMIT/MIT. To investigate whether phenotypes are either retained or changed when recovery time is given or exposure continues, we adopted two types of exposure scenarios: parental generation exposure with recovery of three further generations (parental exposure, PE) and consecutive exposure over four generations (multigenerational exposure, ME). The responses of the daphnids were compared for each generation using reproductive capacity, body size, and morphology endpoints. In the P0 generation, a significant effect of CMIT/MIT was observed on offspring number per female organism (decreased by 34.2%), but not on reproduction time. However, the PE group gradually improved in offspring number (decreased by 23.5% and 1.5%) and showed a delayed first reproduction time (increased by 16.7% and 16.3%, respectively) across generations (Figure 5A,B). In contrast to PE, the most severe effects were manifested in the number of neonates and first reproduction time (decreased by 51.5% and increased by 17.8%, respectively) in the F1 generation of the ME group, whereas beneficial effects (increased by 21.5% and 4.7%, respectively) were observed in the F3 generation (Figure 5).

CMIT/MIT exposure did not affect the body size nor the morphology of daphnids in the parental generation (Figure 3B and Figure S3), but the changes in body size and egg-holding rate after exposure ceased (F1) (Figure 5C,D). Under the PE scenario, the body size of daphnids significantly decreased (by 36.4%) in the unexposed F1 generation and thereafter recovered steadily towards the normal level. This suggests that CMIT/MIT exposure exerts delayed toxicity with respect to the growth endpoint. On the contrary, under the ME scenario, body size was not altered in the F1 generation, and a marked elevation was observed in the F3 generation (20.6%) (Figure 5C). Additionally, the egg-holding rate in the adult brood chamber varied between PE and ME scenarios, and these results accorded with those of body size in each group (Figure 5D).

Under the PE scenario, it is plausible that the effect on the F1 generation was due to the maternal effect [36]. When the embryo undergoes development in the mother’s body, maternal exposure to CMIT/MIT (in our study, P0) could impact the subsequent progeny as embryo (F1) or germ line (F2) [23]. For this reason, obvious transgenerational effects need to be monitored from the F3 generation. Under the PE scenario, maternal effects were shown by the number of neonates and growth in F1, which recovered in F3. However, delays in clutch timing lasted up to the F3 generation, suggesting that CMIT/MIT has a transgenerational effect on reproduction (Figure 5).

The organisms under the ME scenario seem to be acclimated to multigenerational exposure to CMIT/MIT, as their phenotypic indicators showed that exposed daphnids had even faster and higher reproduction and growth rate than the unexposed controls. Adaptation demands heritable changes across generations owing to the modified genome sequence and increases the fitness of populations, whereas acclimation is induced by the rapid defense mechanisms of individuals and changes morphological, physiological, and behavioral traits in response to stress [37]. Since we observed only four generations in the present study, the altered physiological traits under ME might have been due to an acclimatory/defensive response to CMIT/MIT. In previous studies, daphnids exposed to various contaminants, such as organically contaminated stream water [14], cadmium [13], and zinc [38], over multiple generations displayed physiological acclimation for a particular range of concentration. Kim et al. reported that multigenerational exposure to tetracycline can induce reproductive impairment and an increase in somatic growth with the increase in generation number due to a defense mechanism based on the “principle of energy allocation” [39]. However, we could not find the fitness–cost between the two endpoints. Other authors demonstrated that the number of offspring can be governed by maternal body size. For example, toxic chemicals cause the neonates to mature to a smaller size, and a smaller adult subsequently produces fewer eggs [40].

### 3.5. Genotoxic and Epigenotoxic Responses to Parental and Multigenerational Exposure to CMIT/MIT: PE vs. ME

Under the ME scenario, the acclimation of daphnids was suggested after continuous exposure to CMIT/MIT. Under the PE scenario, the maternal effects of CMIT/MIT exposure were observed on the growth and reproduction endpoints. Physiological acclimation can evolve to genetic adaptation when exposure continues [37]. A possible association between epigenetic changes, and the trans- and multigenerational effects of chemical exposure has also been suggested [24]. Maternal exposure to chemicals could affect subsequent unexposed generations by maintaining epigenetic states [23]. To understand the underlying mechanism, we investigated the genotoxicity and epigenotoxicity of CMIT/MIT under PE and ME scenarios.

**Figure 6:**
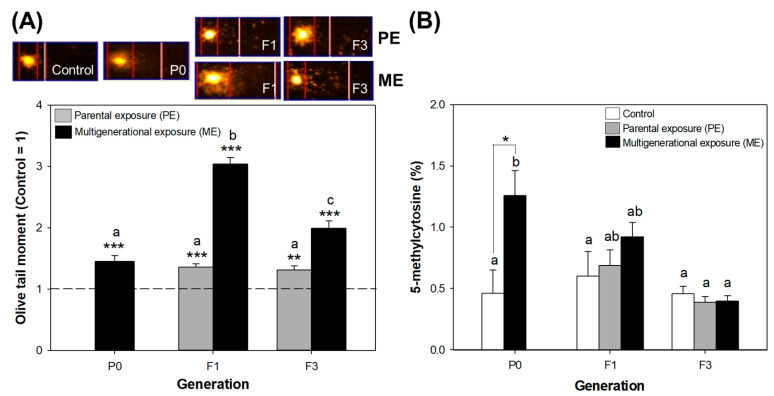
Genotoxic and epigenetic responses of daphnids to parental and multigenerational exposure to EC_20_ CMIT/MIT: (**A**) Comparison of the measured DNA damage with the representative images of tail moment. (**B**) Comparison of % global DNA methylation. Asterisks indicate significant differences between the exposure and control groups: * *p* < 0.05, ** *p* < 0.01, and *** *p* < 0.001. Letters denote the homogeneity between groups (Bonferroni’s test).

Exposure to CMIT/MIT caused damage to the DNA in both exposure scenarios across the generations, with the most severe damage being in F1 under ME (Figure 6A). As CMIT/MIT is electrophilic, its reactive electron-accepting functional groups can react with a wide range of nucleophilic biomolecules [41]. This mechanism might lead to DNA damage in daphnids exposed to CMIT/MIT.

DNA damage can be sufficiently accumulated or sustained in *D. magna* due to continuous exposure to stresses [42,43,44]. The most interesting finding is that DNA damage in the PE group was steadily higher than that in the control group, even in unexposed populations (F1 and F3) (Figure 6A). There was a slight decline from P0 to F3, and this was in line with recovery in reproduction (Figure 5A). Though the present study could not provide a clear explanation, failure to repair DNA might lead to continuous DNA damage in unexposed generations. Atienzar and Jha demonstrated that some of benzo[a]pyrene-induced DNA alterations were transmitted from the mother to unexposed offspring, and some of others were fully repaired or reversed [45].

On the other hand, the ME population showed cumulative effects in F1 and acclimated traits to CMIT/MIT, with a significant decrease in DNA damage from the F1 generation to the F3 generation (Figure 6A). Additionally, the profound DNA damage in F1 in ME might be one of the causes of severe reproductive failure (Figure 5A), and it could be related to the downregulation of vitellogenin fused with superoxide dismutase, considering the proteomic results of the P0 generation. However, our results do not indicate a clear correlation between DNA damage and reproduction spanning all generations, which could be because DNA damage is more sensitive than effects at the individual level, such as growth and reproduction [43,45,46].

The contaminant-associated alterations in global levels of DNA methylation can be useful in identifying the potential of chemicals to influence epigenetic processes or functions, although it does not sufficiently represent the possibility of gene-specific changes [47]. We speculated that changes in DNA methylation might be persistent because phenotypic changes in reproduction and DNA damage were retained up to the F3 generation. However, CMIT/MIT exposure in P0 led to a significant increase in global DNA methylation, and those became normal levels in the F3 generations under both scenarios, as shown in Figure 6B. Increased DNA methylation in the P0 generation was alleviated in F1, but its levels in the ME and PE groups were still higher than in the control group. Epigenetic marks can broadly and rapidly arise within a single generation of a population in response to environmental stressors, and they can also be lost after returning to favorable environmental conditions [20]. After CMIT/MIT exposure ceased, DNA methylation in the population washed out in the PE scenario. However, increased DNA methylation went back to normal levels in F1 and F3 even in the ME scenario, where daphnids continued to be exposed to CMIT/MIT. This result may be possible due to the acclimation of organisms under continuous multigenerational exposure.

Overall, our results are as follows:

1. CMIT/MIT exposure caused deleterious effects on reproduction, and the proteomic analyses suggested that reproductive failure may have been due to the decreased expression of vitellogenin-related proteins in *D. magna*.
2. Parental exposure to CMIT/MIT (PE) caused transgenerational effects on the time to first reproduction (F3) and parental effects (F1) on reproductive capacity and growth recovered after the termination of exposure (F3).
3. Multigenerational exposure to CMIT/MIT (ME) caused an accumulative adverse effect on reproduction (F1), while acclimatory/defensive responses were observed under continued chemical exposure (F3).
4. DNA damage was sustained and then decreased over generations in the ME scenario, indicating that DNA damage might be associated with reproductive toxicity and acclimatory/defensive responses to chemical exposure.
5. DNA methylation increased in daphnids exposed to CMIT/MIT in P0, but it washed out across the subsequent generations under both the PE and ME scenarios. Namely, genotoxicity had a closer association with the inheritance of modified phenotypes, particularly reproduction, than epigenotoxicity.

## 4. Conclusions

We found that CMIT/MIT exposure caused serious reproductive toxicity in *D. magna* at relatively low concentrations that aquatic systems could be exposed to. Multigenerational observations revealed parental effects (F1) and subsequent recovery (F3) under the parental exposure (PE) scenario in addition to cumulative effects (F1) and acclimatory/defensive traits (F3) under the multigenerational exposure (ME) scenario. Further analysis of gene-specific DNA methylation related to reproduction and DNA damage repair is required to fully understand the relationship between CMIT/MIT-induced multigenerational reproductive toxicity and epigenetic changes in daphnids. However, this study provides hazard information on CMIT/MIT with respect to multifaceted endpoints, including proteomic alteration, DNA damage, and DNA methylation. Our results also emphasize that the observation of toxicity within a single generation is not sufficient for reflecting environmentally relevant exposures; thus, the consideration of exposure duration and generations should be applied to the evaluation of ecotoxicity and the risk management of isothiazolinone biocides.

## Figures and Tables

**Table 1:** Nominal concentration of CMIT/MIT mixture and concentrations of CMIT and MIT measured with HPLC. CMIT/MIT mixture solutions were diluted in M4 medium and measured after 7 days. The measured concentrations are presented as means ± SD.

Nominal Concentration (mg/L)		Measured Concentration (mg/L)	
CMIT/MIT Mixture (3:1)		Day 0	Day 7
Standard solution	14,000	-	CMIT: 11,300 MIT: 3000
Stock solutions	1.4	CMIT: 0.99 ± 0.17 MIT: 0.35 ± 0.06	CMIT: 1.03 ± 0.24 MIT: 0.37 ± 0.04
	0.14	CMIT: 0.10 ± 0.01 MIT: 0.04 ± 0.01	CMIT: 0.13 ± 0.02 MIT: 0.04 ± 0.01
Detection limit (mg/L)		≥CMIT: 0.100, MIT: 0.030	

## Data Availability

Proteomic data are available in the Supplementary Materials. Data, associated metadata, and calculation tools are available from the corresponding author (jinhchoi@uos.ac.kr).

## Supplementary Materials

The following supporting information can be downloaded at: https://www.mdpi.com/article/10.3390/toxics11040388/s1, Figure S1: The experimental setup of live imaging for behavior assay, Figure S2: First reproduction time of daphnids exposed to CMIT/MIT, Figure S3: Morphological observation of 7-day-old daphnids exposed to EC_20_ CMIT/MIT, Table S1: High-performance liquid chromatography with diode array detection (HPLC-DAD) conditions used in this study, Table S2: Detected concentrations of CMIT and MIT in products and aquatic environments, Table S3: Acute lethal concentrations (LCs) and chronic effective concentrations (ECs) of CMIT/MIT in *Daphnia magna*, Table S4: Differentially expressed proteins (DEPs) after exposure to EC_20_ CMIT/MIT compared with the control group in the parental generation (P0) of *Daphnia magna*. Refs [48,49,50] has been listed.
