# Supplementary material for: Trans- and Multigenerational Effects of Isothiazolinone Biocide CMIT/MIT on Genotoxicity and Epigenotoxicity in Daphnia magna
Source: Toxics. 2023 Apr 20;11(4):388. doi: 10.3390/toxics11040388 (PMC10140887; doi:10.3390/toxics11040388)
Supplement: Supplementary file 1 [file toxics-11-00388-s001.zip › toxics-2315463-supplementary.pdf]

Supporting Information

Trans- and multigenerational effects of isothiazolinone biocide CMIT/MIT on genotoxicity and epigenotoxicity in *Daphnia magna*

Jiwan Kim and Jinhee Choi*

School of Environmental Engineering, University of Seoul, 163 Seoulsiripdae-ro, Dongdaemun-gu, Seoul 02504, Republic of Korea

*Corresponding author:

Tel: 82-2-6490-2869

Fax: 82-2-6490-2859

E-mail: jinhchoi@uos.ac.kr

Abbreviations

CMIT/MIT: The mixture of 5-Chloro-2-methyl-3(2H)-isothiazolone and 2-methyl-3(2H)-isothiazolone

PE: Parental exposure

ME: Multigenerational exposure

HPLC-DAD: High-performance liquid chromatography with diode array detection

Summary of supplementary materials:

Sixteen pages

Supplementary method

Three figures

Four tables

References

Table of Contents

1. Supplementary method

2. Supplementary figures

Figure S1. The experimental setup of live imaging for behavior assay.

Figure S2. First reproduction time of daphnids exposed to CMIT/MIT.

Figure S3. Morphological observation of 7-day-old daphnids exposed to EC_{20} CMIT/MIT.

3. Supplementary tables

Table S1. High-performance liquid chromatography with diode array detection (HPLC-DAD) conditions used in this study.

Table S2. Detected concentrations of CMIT and MIT in products and aquatic environments.

Table S3. Acute lethal concentrations (LCs) and chronic effective concentrations (ECs) of CMIT/MIT in *Daphnia magna*.

Table S4. Differentially expressed proteins (DEPs) after exposure to EC_{20} CMIT/MIT compared with the control group in the parental generation (P0) of *Daphnia magna.*

1. Supplementary method

Quantitative MS-based proteomics

For proteomic analysis, proteins from *Daphnia magna* were lysed in RIPA buffer (Thermo Fisher Scientific Inc., Rockford, IL) with sonication at 30% for 30 seconds. Extracted proteins were precipitated in 10% trichloroacetic acid overnight at 4 °C to remove the detergent and were centrifuged at 14,000xg for 10 min at 4 °C. The pellets were rinsed with ice-cold acetone twice and recentrifuged at 14,000xg for 10 min at 4 °C. Protein pellets were dissolved in 50 mM ammonium bicarbonate (ABC) buffer. For reduction and alkylation, the proteins were supplemented with 15 mM dithiothreitol in 20 mM ABC at 56 °C for 30 min and alkylated using iodoacetamide in 25 mM ABC at room temperature for 30 min under dark conditions. To withdraw the reduction and alkylation reagents, the proteins were subjected to ice-cold acetone precipitation at 20 °C for 4 h. After centrifugation at 14,000xg for 10 min at 4 °C, the protein pellets were re-suspended in 100 mM triethylammonium bicarbonate and analyzed with a BCA protein assay kit (Thermo Fisher Scientific Inc., Rockford, IL).

For the quantitative proteomic approach, each 100 mg of proteins was transferred to new Eppendorf tubes and incubated at 37 °C for 16 h with trypsin at 1:50 w/w to make peptides. Then, 0.8 mg of sixplex tandem mass tag (TMT) reagent (Thermo Fisher Scientific Inc., Rockford, IL) was added to 41 mL of anhydrous acetonitrile in each tube. Additionally, peptides were labeled with TMT at room temperature for an hour. For the quenching reaction, peptides were supplemented with 8 mL of 5% hydroxylamine and incubated for 15 min. Finally, the labeled peptides were combined, and fractionation steps were conducted using high-pH reversed-phase peptide fractionation kits (Thermo Fisher Scientific Inc., Rockford, IL), following the procedure provided by the product's manufacturer. Before the mass spectrometry analysis, all samples were desalted with a C18 Zip tip (Merck Millipore, Billerica, MA) according to the manufacturer's instructions and dried completely using a speed vacuum.

Dried peptides were dissolved in solution A (water with 0.1% formic acid) and analyzed using a nano LC-MS/MS system consisting of Easy-nLC 1000 (Thermo Fisher Scientific Inc., Waltham, MA) and a Q Exactive Hybrid Quadrupole-Orbitrap mass spectrometer (Thermo Fisher Scientific Inc., Waltham, MA). The separation of peptides was performed using a home-made C18 reversed-phase column (Dr. Maisch GmbH, Ammerbuch-Entringen, Germany) with a nanospray source. A 60 min linear gradient was used at a flow rate of 200 nL/min from 2 to 20% with solution B (0.1% formic acid in 100% acetonitrile) for 44 min, 20-90% with solution B for 5 min, and at 90% with solution B for over 11 min to completely elute the peptides. Q-Exactive was operated in a data-dependent mode using the following parameters: nanospray voltage with positive charge of 2.2 KV; scan range of 300 to 1400 m/z; a total of 15 data-dependent higher-energy collisional dissociation MS/MS scans per full MS; resolution of 70,000 and 17,500 for full MS and MS/MS, respectively; dynamic exclusion time of fragmented ions of 25 seconds; AGC target of 3e6; maximum inject time for full MS of 50 ms. Experiments were performed with biological duplicates and technical duplicates.

To identify and quantify the proteomic results, raw files were merged using MM file conversion and searched using Mascot 2.3 (Matrix Science) with the *Daphnia magna* and *Daphnia pulex* proteome database downloaded from Uniprot (http://www.uniprot.org/). The search parameters were as follows: two missed trypsin cleavages; fixed modification of carbamidomethylation to cysteine; variable modification of oxidation to methionine and

protein N-term acetylation. MS and MS/MS tolerance were 10 ppm and 0.05 Da, respectively. The search results were filtered with a mascot score ≥ 20 and a false discovery rate (FDR) ≤ 0.01.

2. Supplementary figures

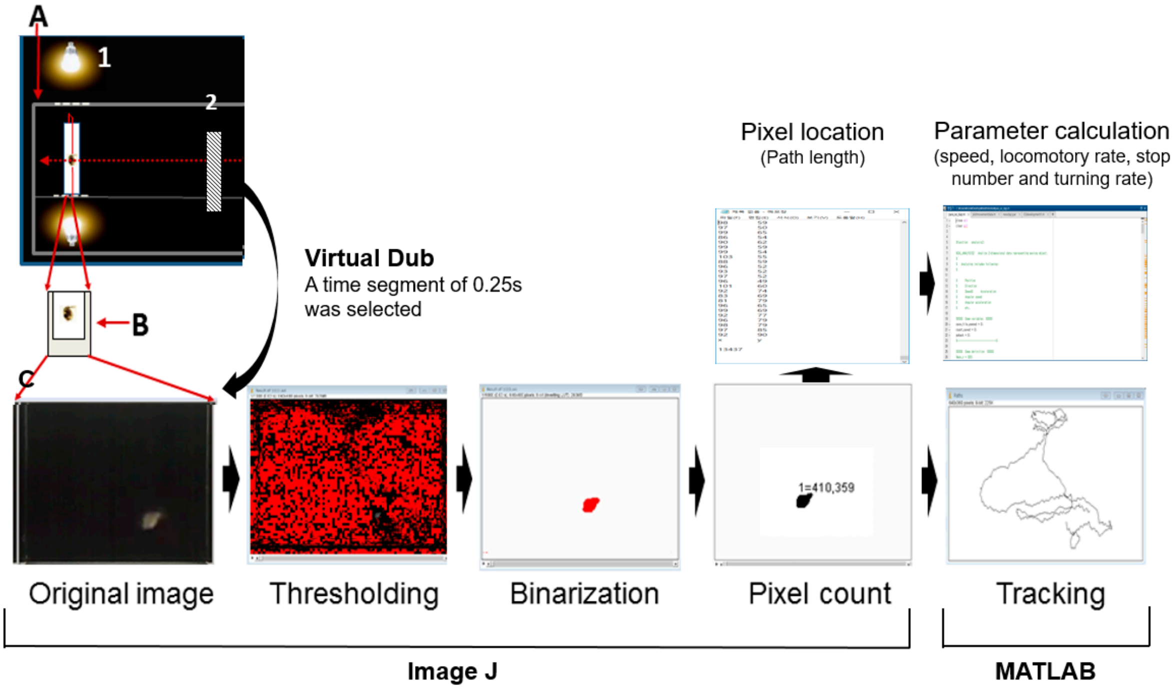

Figure S1. The experimental setup of live imaging for behavior assay. (A) Camera set (300 X 180 X 240 mm) with black sheet for the background of the image: 1. Flash to uniformly scatter the light from two sides of the camera set; 2. camera (iphone3gs). (B) Chamber (100 X 30 X 95 mm) with medium (M4 or CMIT/MIT) and one daphnia in the well. (C) Procedures of image processing in Image J and MATLAB. The original images that were obtained from the chamber were binarized with an optimized threshold, and the x, y coordinates of the object (pixel) were counted so that the tracking analysis could be used for illustrating swimming behaviors such as speed, turning, and hop and sink.

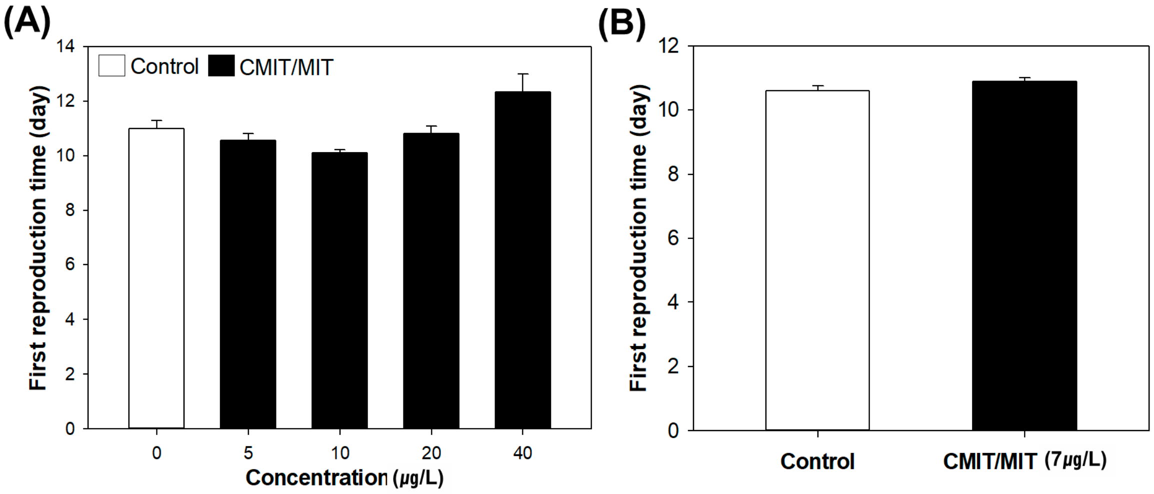

Figure S2. First reproduction time of daphnids exposed to CMIT/MIT. (A) First reproduction time of daphnids exposed to 5, 10, 20, and 40 μg/L CMIT/MIT, except for the highest concentration, at which adults never produced neonates. (B) First reproduction time of daphnids after exposure to EC_{20} CMIT/MIT (7 μg/L).

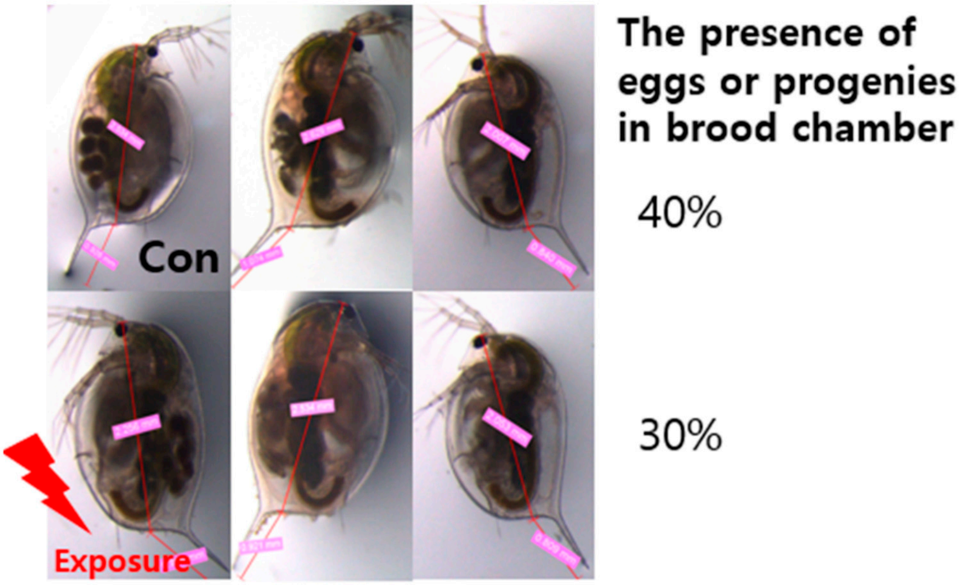

Figure S3. Morphological observation of 7-day-old daphnids exposed to EC_{20} CMIT/MIT. % indicates the percentage of organisms that had eggs or progenies in their brood chamber.

2. Supplementary tables

Table S1. High-performance liquid chromatography with diode array detection (HPLC-DAD) conditions used in this study.

Analytical conditions of HPLC-DAD			
Chemical	CMIT and MIT		
Sample pretreatment	Filtration of samples with 0.45 μm nylon filter		
Model	Agilent 1260 Infinity HPLC-DAD		
Column	Eclipse XDB-C18 (4.6 mm*250 mm*5 μm)		
Column temperature	30 ℃		
Detection wavelength	280 nm		
Flow rate	1.0 mL/min		
Injection volume	10 μL		
Mobile phase	A: 0.1% formic acid in water, B: acetonitrile		
Gradient	Time (min)	A (%)	B (%)
	0.00	95	5
	3.00	95	5
	8.00	80	20
	9.00	95	5
	21.00	95	5

Table S2. Detected concentrations of CMIT and MIT in products and aquatic environments.

Sample		Detection Instrument	Detected Concentration or Amount	Reference
Product	Three nail polishes from commercial markets	UPLC-MS/MS	CMIT: 17.19-35.24 μg/g<br>MIT: 11.45-34.63 μg /g	Lee et al., 2020
	Cosmetics (rinse-off and leave-on types) and household product samples (detergents and cleaning products)	HPLC-MS/MS	CMIT/MIT mixture (3:1): 0.000095-0.0067% (w/w) in cosmetic samples and 0.0006-0.0011% (w/w) in home care products	Alvarez-Rivera et al., 2012
	Water-based adhesive	HPLC-MS/MS	CMIT: 3.28-28.36 mg/kg<br>MIT: 12.94-60.79 mg/kg	Zhong et al., 2019
	Cosmetics	HPLC	CMIT: 3.40-8.59 μg/g	Baranowska and Wojciechowska, 2013
Environment	Surface water, dry and wet atmospheric depositions, wastewater treatment plant (WWTP) influent and effluent, and storm water (in Paris)	HPLC-MS/MS	1) Concentrations in dissolved fractions<br>CMIT: 5.6-120 ng/L<br>MIT: 14-860 ng/L<br>2) Concentrations in particulate fractions<br>CMIT: 32-4200 ng/g<br>MIT: 6.4-360 ng/g<br>3) Total concentration<br>CMIT: 5.6-57 ng/L<br>MIT: 39-106 ng/L	Paijens et al., 2020
	Environmental water samples (in Poland)	HPLC	CMIT: 5.72-11.57 μg/L in river	Baranowska and Wojciechowska, 2013
	Combined sewer overflows (CSOs), and WWTP influents and effluents	HPLC-MS/MS	1) CSOs<br>CMIT: <9.5-160 ng/L<br>MIT: 9.8-290 ng/L<br>2) WWTP influents<br>CMIT: <13 ng/L<br>MIT: 0.35-0.86 μg/L<br>3) WWTP effluents<br>CMIT: <3.7 ng/L<br>MIT: 0.039-0.35 μg/L	Paijens et al., 2021
	Beach sand, soil, and untreated sewage	LC-MS/MS	1) Beach sands<br>MIT: 2.19-4.48 μg/L<br>2) Soils<br>MIT: 1.04-10.8 μg/L<br>4) Untreated sewage<br>MIT: 1.21 μg/L	Nowak et al., 2020
	Urban surface waters and storm water sewers (in Denmark)	HPLC-MS/MS	1) Urban surface water<br>MIT : 40-50 ng/L<br>2) Storm water (catchment)<br>MIT: 1.6 mg/rain event	Bester et al., 2014

Table S3. Acute lethal concentrations (LCs) and chronic effective concentrations (ECs) of CMIT/MIT in *Daphnia magna*. Acute LCs and chronic ECs were estimated using drc package in R, and 95% confidence levels were marked together.

LC/EC	Acute Assay (Mortality)	Chronic Assay (Reproduction)
	Estimated Lethal Concentrations (μg/L)	Estimated Effective Concentrations (μg/L)
10	17.74<34.79<51.83	1.92<4.25<6.58
20	27.50<43.36<59.23	3.71<6.44<9.17
50	48.30<63.20<78.09	9.57<13.08<16.60
90	63.72<114.81<165.90	24.59<40.25<55.90

Table S4. **Differentially expressed proteins (DEPs) after exposure to EC_{20} CMIT/MIT compared with the control group in the parental generation (P0) of *Daphnia magna*.** The upregulated DEPs (fold change >1.20) and downregulated DEPs (fold change <0.83) are listed.

Protein ID	Protein Name	Gene Name	Fold Change
**Upregulated Proteins**			
A0A0P6A1T0	Putative endocuticle structural glycoprotein SgAbd-1	APZ42_029388	1.99583
E9G2A6	AA_TRNA_LIGASE_II domain-containing protein	DAPPUDRAFT_127463	1.89533
E9G2V0	Uncharacterized protein	DAPPUDRAFT_313111	1.53482
E9G6X0	Cytochrome c oxidase subunit	DAPPUDRAFT_230594	1.50628
E9GF22	Uncharacterized protein	DAPPUDRAFT_230815	1.47861
E9G914	Uncharacterized protein	DAPPUDRAFT_194717	1.44289
E9HEE5	Uncharacterized protein	DAPPUDRAFT_202596	1.43802
E9HRW4	Uncharacterized protein	DAPPUDRAFT_333111	1.42743
A0A164NMQ0	Arginine kinase	APZ42_030553	1.42102
A0A0N8A729	Cuticle protein (Cuticular protein)	APZ42_016420	1.41727
E9GHL7	Uncharacterized protein	DAPPUDRAFT_303663	1.41615
A0A164PIN1	Endocuticle structural glycoprotein SgAbd-1	APZ42_029384	1.40575
A0A164KJQ5	VWFD domain-containing protein	APZ42_033978	1.40106
E9FRZ6	Uncharacterized protein	DAPPUDRAFT_230081	1.38788
A0A162BZC1	Putative endocuticle structural glycoprotein SgAbd-1 (fragment)	APZ42_004954	1.38053
E9GHL2	Uncharacterized protein	DAPPUDRAFT_303661	1.37861
E9H6I9	Transgelin	DAPPUDRAFT_308120	1.36334
A0A164LPA0	Uncharacterized protein	APZ42_032543	1.35986
E9GA32	Uncharacterized protein	DAPPUDRAFT_301581	1.35907
A0A162NYZ9	Putative aminopeptidase N	APZ42_014873	1.35493
E9HPM3	Uncharacterized protein	DAPPUDRAFT_302740	1.35125
E9H2T2	Uncharacterized protein	DAPPUDRAFT_200339	1.33574
E9GK07	Uncharacterized protein	DAPPUDRAFT_212175	1.33455
A0A164YKH6	Putative basement membrane-specific heparan sulfate proteoglycan core protein	APZ42_019106	1.32984
E9GQU4	C-type lectin domain-containing protein	DAPPUDRAFT_305228	1.32859
A0A0P5MYN2	Myosin regulatory light-chain 2 smooth muscle (myosin regulatory light polypeptide 9)	APZ42_011326	1.32682
A0A162S9F3	Chitin-binding type-2 domain-containing protein	APZ42_012228	1.32342
E9GHT0	Uncharacterized protein	DAPPUDRAFT_303748	1.31695

A0A164H442	Lysosomal alpha-glucosidase (fragment)	APZ42_004352	1.31549
A0A164IVB1	Uncharacterized protein	APZ42_001606	1.30587
E9GHL1	Uncharacterized protein	DAPPUDRAFT_303723	1.30421
E9HAU2	Uncharacterized protein (fragment)	DAPPUDRAFT_60605	1.30329
A0A162BSN0	Cuticular protein (Fragment)	APZ42_008427	1.29376
E9HRW3	Uncharacterized protein	DAPPUDRAFT_333110	1.2929
E9FX34	Nucleoplasmin domain-containing protein	DAPPUDRAFT_305495	1.2894
A0A162DGC9	Myosin heavy chain	APZ42_024143	1.27965
E9G583	Uncharacterized protein	DAPPUDRAFT_313764	1.27541
E9FV57	Uncharacterized protein	DAPPUDRAFT_220693	1.26763
E9G2T7	Anion exchange protein	DAPPUDRAFT_193096	1.26489
E9FRT1	Muscle-specific actin	ACT2A	1.26457
E9G620	Uncharacterized protein	DAPPUDRAFT_230573	1.26047
A0A164PJ70	Cuticular protein 49Ag	APZ42_029383	1.2601
E9G9M2	Uncharacterized protein	DAPPUDRAFT_301619	1.25948
E9FUF6	AA_TRNA_LIGASE_II_ALA domain-containing protein	DAPPUDRAFT_191289	1.25742
A0A164PJ82	Cuticular protein 49Ag	APZ42_029380	1.25574
E9FSI1	Uncharacterized protein	DAPPUDRAFT_190431	1.25402
A0A0P5GEN8	Paramyosin (Paramyosin, long form)	APZ42_025173	1.2499
A0A164M3U2	Villin-1	APZ42_032294	1.24151
A0A164NU47	Vitellogenin domain-containing protein	APZ42_030366	1.24042
A0A164IVC2	Uncharacterized protein (fragment)	APZ42_001605	1.23952
A0A162RAN8	Angiotensin-converting enzyme (EC 3.4.-.-)	APZ42_012971	1.23851
A0A164RZD6	Mitochondrial-processing peptidase subunit beta	APZ42_026803	1.23681
A0A164XNK5	Chymotrypsin-like protein	APZ42_019721	1.23599
E9HPM5	Uncharacterized protein	DAPPUDRAFT_332314	1.23232
A0A162NZ08	Endoplasmic reticulum aminopeptidase 1	APZ42_014872	1.2298
A0A164VQM0	Putative cuticular protein analogous to peritrophins 3-C	APZ42_022677	1.22849
E9GLD2	Malic enzyme	DAPPUDRAFT_304467	1.22659
A0A0P5ZXT5	Di-domain hemoglobin	APZ42_022504	1.22463
E9FTM4	Peptidase S1 domain-containing protein	DAPPUDRAFT_230174	1.22109
E9HAW7	Sodium/potassium-transporting ATPase subunit alpha	DAPPUDRAFT_309219	1.21996
A0A162Q5W2	Glutathione S transferase E10	APZ42_014224	1.21975

	(glutathione S-transferase delta/epsilon10 isoform a)		
A0A164X3C5	NAD(P) transhydrogenase, mitochondrial	APZ42_020909	1.21751
A0A0P5M8I8	ATP synthase subunit gamma	APZ42_021777	1.21281
E9FVX8	Lectin, mannose-binding, 1	DAPPUDRAFT_191157	1.21088
A0A164WWC9	Uncharacterized protein	APZ42_021013	1.21056
E9GGJ0	Peptidase S1 domain-containing protein	DAPPUDRAFT_230857	1.20972
E9FWY0	Tubulin beta chain	DAPPUDRAFT_305638	1.20515
A0A162QYY2	Putative basement membrane-specific heparan sulfate proteoglycan core protein	APZ42_013388	1.20411
A0A164RDZ2	Tissue factor pathway inhibitor	APZ42_027182	1.20168
**Downregulated Proteins**			
A0A164HC59	Vitellogenin fused with superoxide dismutase (fragment)	APZ42_003944	0.45368
E9HF43	Uncharacterized protein	DAPPUDRAFT_202746	0.47291
A0A162C7M7	Vitellogenin fused with superoxide dismutase (fragment)	APZ42_001431	0.51557
A0A164EIE6	Vitellogenin fused with superoxide dismutase (fragment)	APZ42_008638	0.52241
A0A164DXH9	Vitellogenin domain-containing protein (fragment)	APZ42_009585	0.62472
A0A164H2H0	Uncharacterized protein (fragment)	APZ42_004430	0.64195
A0A164I1F8	Vitellogenin domain-containing protein (fragment)	APZ42_002789	0.64997
A0A164ISY5	Vitellogenin fused with superoxide dismutase (fragment)	APZ42_001711	0.65446
A0A164F1A5	Vitellogenin domain-containing protein (fragment)	APZ42_007869	0.66318
E9GVW1	Uncharacterized protein	DAPPUDRAFT_213992	0.67158
A0A164EGF3	Uncharacterized protein (fragment)	APZ42_008709	0.67645
E9GEV7	Uncharacterized protein	DAPPUDRAFT_302797	0.6969
E9HK57	Uncharacterized protein	DAPPUDRAFT_189509	0.7017
A0A0P5VG56	Pyruvate dehydrogenase E1 component subunit alpha (EC 1.2.4.1)	APZ42_016439	0.70586
E9G7B4	Uncharacterized protein	DAPPUDRAFT_314742	0.70803
E9GMY5	Uncharacterized protein	DAPPUDRAFT_128589	0.71299
A0A164H8I7	DUF1943 domain-containing protein	APZ42_004153	0.71447
A0A162NI02	Cellobiohydrolase CHBI	APZ42_015977	0.71553
E9GA59	Uncharacterized protein	DAPPUDRAFT_230663	0.71659

A0A164HTE3	Vitellogenin fused with superoxide dismutase (fragment)	APZ42_003122	0.72408
E9H169	Ribosomal_L16 domain-containing protein	DAPPUDRAFT_231299	0.73518
E9G3M6	Uncharacterized protein	DAPPUDRAFT_230521	0.74254
A0A0N8E998	60S ribosomal protein L35a	APZ42_017722	0.74308
E9H224	Cct5-prov protein	DAPPUDRAFT_200182	0.74495
E9H3D5	Uncharacterized protein	DAPPUDRAFT_231341	0.74711
A0A164IW71	VWFD domain-containing protein (Fragment)	APZ42_001569	0.74729
E9GF14	C-type lectin domain-containing protein	DAPPUDRAFT_230812	0.75609
A0A164PP21	60S ribosomal L22 1-like protein	APZ42_029533	0.75728
E9G7M2	Proteasome subunit beta (EC 3.4.25.1)	DAPPUDRAFT_99667	0.76059
E9HC90	Proliferating cell nuclear antigen	DAPPUDRAFT_309373	0.76553
A0A164TME0	Uncharacterized protein	APZ42_024847	0.76749
E9GFW1	Uncharacterized protein	DAPPUDRAFT_303327	0.77115
E9G2H9	40S ribosomal protein S6	Rps6	0.77583
E9H405	Malectin domain-containing protein	DAPPUDRAFT_307649	0.78648
E9GXS2	KH type-2 domain-containing protein	DAPPUDRAFT_306633	0.78779
E9HZI6	Vitellogenin fused with superoxide dismutase	VTG1	0.78985
E9FUS8	Histone H3	DAPPUDRAFT_235586	0.79213
E9GYM5	T-complex protein 1 subunit gamma	DAPPUDRAFT_306806	0.79262
E9HJW4	Ribosomal protein L19	DAPPUDRAFT_301730	0.79294
E9FY06	Uncharacterized protein	DAPPUDRAFT_305566	0.79622
E9HJ97	Ribosomal_S7 domain-containing protein	DAPPUDRAFT_301516	0.79677
E9HAP6	Small nuclear ribonucleoprotein Sm D2 (Sm-D2) (snRNP core protein D2)	DAPPUDRAFT_309154	0.79888
A0A162PJL4	60S ribosomal protein L13	APZ42_015493	0.80147
E9HNC6	HATPase_c domain-containing protein	DAPPUDRAFT_302452	0.80185
E9HU52	Uncharacterized protein	DAPPUDRAFT_231884	0.80305
E9FSQ9	Uncharacterized protein	DAPPUDRAFT_310174	0.80394
E9HEA0	Uncharacterized protein	DAPPUDRAFT_300630	0.80704
E9G788	Uncharacterized protein	DAPPUDRAFT_301074	0.81083
E9FR40	40S ribosomal protein S12	DAPPUDRAFT_230117	0.8112
E9GAT4	Protein transport protein Sec61 subunit beta	DAPPUDRAFT_230689	0.81544
E9G8G7	Uncharacterized protein	DAPPUDRAFT_223052	0.81955

A0A0P6GST7	116 kDa U5 small nuclear ribonucleoprotein component protein (elongation factor G, mitochondrial)	APZ42_020697	0.82059
E9H8K5	Uncharacterized protein	DAPPUDRAFT_308675	0.8215
A0A164YQP7	Thioredoxin domain-containing protein	APZ42_018662	0.82263
E9HDC8	Ribosomal_S10 domain-containing protein	DAPPUDRAFT_300540	0.82303
E9HTS2	Uncharacterized protein	DAPPUDRAFT_231875	0.82532
A0A164L230	Cathepsin L	APZ42_033465	0.82705
E9H9Z6	Ribosomal_S13_N domain-containing protein	DAPPUDRAFT_308825	0.82899
E9FU59	40S ribosomal protein S3a	DAPPUDRAFT_303071	0.83001
E9G121	Uncharacterized protein	DAPPUDRAFT_307788	0.83015
E9FYC3	Uncharacterized protein	DAPPUDRAFT_306375	0.83103
E9GGM3	40S ribosomal protein S8	DAPPUDRAFT_303431	0.83117
E9HBZ4	Uncharacterized protein	DAPPUDRAFT_256736	0.83181

Reference

Alvarez-Rivera, G., Dagnac, T., Lores, M., Garcia-Jares, C., Sanchez-Prado, L., Lamas, J.P., Llompart, M., 2012. Determination of isothiazolinone preservatives in cosmetics and household products by matrix solid-phase dispersion followed by high-performance liquid chromatography-tandem mass spectrometry. J. Chromatogr. A 1270. https://doi.org/10.1016/j.chroma.2012.10.063

Baranowska, I., Wojciechowska, I., 2013. The determination of preservatives in cosmetics and environmental waters by HPLC. Polish J. Environ. Stud. 22.

Bester, K., Vollertsen, J., Bollmann, U.E., 2014. Water driven leaching of biocides from paints and renders (Pesticide Research No. 156, 2014).

Lee, J.H., Paek, J.H., Park, H.N., Park, S., Kang, H., 2020. Screening and detection of methylisothiazolinone and chloromethylisothiazolinone in cosmetics by UPLC-MS/MS. Anal. Sci. Technol. 33. https://doi.org/10.5806/AST.2020.33.3.125

Nowak, M., Zawadzka, K., Lisowska, K., 2020. Occurrence of methylisothiazolinone in water and soil samples in Poland and its biodegradation by Phanerochaete chrysosporium. Chemosphere 254. https://doi.org/10.1016/j.chemosphere.2020.126723

Paijens, C., Bressy, A., Frère, B., Tedoldi, D., Mailler, R., Rocher, V., Neveu, P., Moilleron, R., 2021. Urban pathways of biocides towards surface waters during dry and wet weathers: Assessment at the Paris conurbation scale. J. Hazard. Mater. 402. https://doi.org/10.1016/j.jhazmat.2020.123765

Paijens, C., Frère, B., Caupos, E., Moilleron, R., Bressy, A., 2020. Determination of 18 Biocides in Both the Dissolved and Particulate Fractions of Urban and Surface Waters by HPLC-MS/MS. Water. Air. Soil Pollut. https://doi.org/10.1007/s11270-020-04546-6

Zhong, H., Li, Z., Chen, S., Zeng, Ying, Zheng, J., Zeng, You, Li, D., 2019. Simultaneous quantitative analysis of six isothiazolinones in water-based adhesive used for food contact materials by high-performance liquid chromatography–tandem mass spectrometry (HPLC–MS/MS). Molecules 24. https://doi.org/10.3390/molecules24213894
